# Cardiolipin Membranes Promote Cytochrome *c* Transformation of Polycyclic Aromatic Hydrocarbons and Their In Vivo Metabolites

**DOI:** 10.3390/molecules29051129

**Published:** 2024-03-03

**Authors:** João Lopes, Dorinda Marques-da-Silva, Paula A. Videira, Alejandro K. Samhan-Arias, Ricardo Lagoa

**Affiliations:** 1 School of Technology and Management, Polytechnic Institute of Leiria, Morro do Lena-Alto do Vieiro, 2411-901 Leiria, Portugal; joao.m.lopes@ipleiria.pt (J.L.); dorinda.silva@ipleiria.pt (D.M.-d.-S.); 2 Laboratory of Separation and Reaction Engineering-Laboratory of Catalysis and Materials (LSRE-LCM), School of Management and Technology, Polytechnic Institute of Leiria, 2411-901 Leiria, Portugal; 3 Associate Laboratory in Chemical Engineering (ALiCE), Faculty of Engineering, University of Porto, Rua Dr. Roberto Frias, 4200-465 Porto, Portugal; 4 Applied Molecular Biosciences Unit (UCIBIO), NOVA School of Science and Technology, NOVA University of Lisbon, 2829-516 Caparica, Portugal; p.videira@fct.unl.pt; 5 Institute for Health and Bioeconomy (i4HB), NOVA School of Science and Technology, NOVA University of Lisbon, 2829-516 Caparica, Portugal; 6 Department of Biochemistry, Autonoma University of Madrid (UAM), C/Arturo Duperier 4, 28029 Madrid, Spain; alejandro.samhan@uam.es; 7 Institute for Biomedical Research ‘Sols-Morreale’ (CSIC-UAM), C/Arturo Duperier 4, 28029 Madrid, Spain

**Keywords:** environmental toxicology, hemeproteins, ionization potential threshold, lipid membrane, mutagenesis, oxidative damage, peroxidases, pollutants, pseudo-peroxidases, radical cations

## Abstract

The catalytic properties of cytochrome *c* (C*c*) have captured great interest in respect to mitochondrial physiology and apoptosis, and hold potential for novel enzymatic bioremediation systems. Nevertheless, its contribution to the metabolism of environmental toxicants remains unstudied. Human exposure to polycyclic aromatic hydrocarbons (PAHs) has been associated with impactful diseases, and animal models have unveiled concerning signs of PAHs’ toxicity to mitochondria. In this work, a series of eight PAHs with ionization potentials between 7.2 and 8.1 eV were used to challenge the catalytic ability of C*c* and to evaluate the effect of vesicles containing cardiolipin mimicking mitochondrial membranes activating the peroxidase activity of C*c*. With moderate levels of H_2_O_2_ and at pH 7.0, C*c* catalyzed the oxidation of toxic PAHs, such as benzo[a]pyrene, anthracene, and benzo[a]anthracene, and the cardiolipin-containing membranes clearly increased the PAH conversions. Our results also demonstrate for the first time that C*c* and C*c*–cardiolipin complexes efficiently transformed the PAH metabolites 2-hydroxynaphthalene and 1-hydroxypyrene. In comparison to horseradish peroxidase, C*c* was shown to reach more potent oxidizing states and react with PAHs with ionization potentials up to 7.70 eV, including pyrene and acenaphthene. Spectral assays indicated that anthracene binds to C*c*, and docking simulations proposed possible binding sites positioning anthracene for oxidation. The results give support to the participation of C*c* in the metabolism of PAHs, especially in mitochondria, and encourage further investigation of the molecular interaction between PAHs and C*c*.

## 1. Introduction

Polycyclic aromatic hydrocarbons (PAHs) constitute a large group of hazardous organic compounds containing two or more fused benzene rings. These compounds emerge in the environment through the release of petroleum products and the incomplete combustion of coal, biomass, and other sources of organic carbon [1,2]. 

Humans are exposed to PAHs through inhalation, ingestion, and skin contact by way of air particulate matter, vehicle exhaust, cigarette smoke, and foods, among other specific exposures [2,3,4,5]. Certain professional groups, such as firefighters and coke oven workers, face elevated PAH exposure [5,6,7].

Epidemiological and experimental studies underscore the association between PAH exposure and pathologies, particularly cancers and cardiovascular diseases [3,8,9,10,11]. Benzo[a]pyrene (BaP) is a recognized carcinogen, whereas other PAHs like naphthalene, anthracene, benzo[a]anthracene (BaA), and benzo[b]fluoranthene (BbF) are classified as potentially carcinogenic to humans [12]. In a recent cohort study, a positive association between dietary PAH intake (BaP, chrysene, BaA, and BbF) and lung/tracheal cancer mortality risk was reported [3]. There are also increasing concerns regarding the neurotoxicity of PAHs, especially in children and occupationally exposed professionals [6,7].

While BaP carcinogenicity is closely related to DNA adduct formation, the overall of toxicity PAHs is associated with oxidative stress, inflammation, mitochondrial damage, and apoptosis [9,13,14,15]. There is growing attention to the involvement of mitochondria in the toxicity of environmental contaminants [8], with studies revealing mtDNA damage, stimulation of mitochondrial apoptosis, and reduced ATP production in animals exposed to BaP and acenaphthene [14,15,16]. Also, mice exposed to BaP showed decreased antioxidants in mitochondria, as well as reduced Krebs cycle enzymes activities, in an organ-independent manner [17]. In cells, low levels of BaP were enough to affect the mitochondrial membrane potential and ATP levels [18]. In other studies, air particulate matter was detected inside cells associated with membrane structures like mitochondria [19], and lipophilic PAHs effectively incorporated into phospholipid membranes [20,21], including those containing cardiolipin (CL), a phospholipid characteristic of bacterial and mitochondrial membranes [22]. Hence, PAHs and their metabolites in cells are probably available to interact with mitochondrial components, especially with membrane-associated lipids and proteins.

The current understanding of the human metabolism of PAHs underlines the contribution of cytochrome P450 (CYP450) monooxygenase or peroxidase activities, combined with epoxide hydrolase and aldo–keto reductase enzymes, in the generation of diverse hydroxylated, epoxide, and quinone PAH derivatives [8,9,21]. Although the precise role of each enzyme in the biotransformation of the different PAHs remains elusive, the toxicological relevance of the in vivo generation of PAH metabolites is well established. Actually, hydroxylated PAHs (HO-PAHs) in urine are commonly used as biomarkers of human PAH exposure [2,5,6,10,11]. The low-molecular-weight metabolites (HO-naphthalene forms) are found at the highest concentrations, and the most abundant is 2-hydroxynaphthalene [2,5,10]. However, the urinary levels of 1-hydroxypyrene among other HO-PAHs were found to be augmented significantly in firefighters after training exercises [5]. Furthermore, hydroxynaphthalenes, hydroxyfluorenes, and 1-hydroxypyrene are the PAH metabolites presenting a closer association to cardiovascular disorders [10,11]. In occupationally exposed individuals, neurological alterations were correlated to the levels of 2-hydroxynaphthalene [6] and 1-hydroxypyrene [7].

In addition to CYP450, several microbial and plant peroxidases were demonstrated to catalyze oxidative modifications of PAHs, attracting interest for their potential ecological and biotechnological relevance [23,24]. However, other hemeproteins like cytochrome *c* (C*c*), more recognized for other biological roles, can also display peroxidase activity, at least under certain activating conditions [24,25]. The pseudo-peroxidase or peroxidase-like activity of C*c* towards organic compounds has been reported [26], including the oxidation of sulfur heterocyclic compounds [27] and PAHs [28]. More specifically, horse C*c* was described to oxidize anthracene, pyrene, and BaP in the presence of 1 mM H_2_O_2_ at pH 6.1 [28]. However, the catalytic abilities of C*c* towards PAHs seem inferior to other peroxidases and CYP450 [29], and do not enable an attack of oxidant-resistant compounds (with very high ionization potential) like phenanthrene and naphthalene [28]. Nevertheless, the catalytic activities measured with anthracene and pyrene suggest that C*c* could oxidize other intermediate resistant PAHs, namely, acenaphthene, contrary to that reported [28]. Moreover, from a toxicological perspective, it is unclear if C*c* can have a significant role in the transformation of PAHs or their metabolites under moderate H_2_O_2_ levels and pHs more representative of biological conditions.

In cells, C*c* is present in the mitochondrial intermembrane space and anchors to the mitochondrial inner membrane, being an essential component of the electron transport chain. In addition, extensive binding to CL in mitochondrial membranes under apoptotic conditions enables the massive release of C*c* into the cytosol, where it can interact with a variety of other targets [30,31]. The CLs (1,3-bis(sn-3′-phosphatidyl)-sn-glycerol) are a group of anionic phospholipids found in the plasma membrane of bacteria and in the inner mitochondrial membranes of eukaryotic cells [30]. Interestingly, exposure to different organic pollutants has caused a huge increase in some CL species in cells [32].

The interaction of C*c* with CL, and the subsequent formation of C*c*-CL complexes, induces structural changes that affect the heme Fe coordination and increase the peroxidase activity of C*c* [30,33,34,35]. Previous work has shown that in the presence of CL-containing phospholipid membranes and sub-millimolar concentrations of H_2_O_2_, the peroxidase activity of C*c* can increase by more than one order of magnitude when measured with standard peroxidase substrates [34,35,36]. Yet, the potential relevance of free or CL-complexed C*c* in the metabolism of environmental toxicants has not been given attention.

C*c* binding to vesicles containing CL, as a model of mitochondrial membranes, was described to decrease the C*c* reduction potential [E^0^ of Fe(III)/Fe(II) redox couple] by about 350–400 mV, to approximately −170 mV [33], which is well within the range of typical peroxidases [23]. For example, horseradish peroxidase (HRP) can oxidize several PAHs to DNA-binding radical cations [37]. Thus, we hypothesized that CL-containing membranes could promote the C*c*-catalyzed transformation of PAHs, and C*c* might participate in the metabolism of these compounds. Preliminary studies with BbF and an azo dye pointed out that CL vesicles indeed accelerated their C*c*-mediated oxidation by H_2_O_2_, as published by our group in a conference abstract [38].

The aims of the present work were to investigate the catalytic ability of C*c* to transform PAHs and metabolites, and to evaluate the effect of CL membranes in that process. For this study, eight PAHs with two to five rings, including compounds of top toxicological relevance, were selected along with two common HO-PAH metabolites. Table S1 of the Supplementary Materials comprises a list of the CAS number, molecular weight, log K_o/w_, water solubility, and ionization potential of the 10 studied compounds.

## 2. Results and Discussion

To investigate the ability of C*c* to transform toxicologically relevant PAHs, we conducted assays at pH 7.0 with a protein concentration of 1 μM, except in the study of comparison to HRP, and always in the presence of H_2_O_2_ at 100 μM, as specified in the Methods Section 3.4. The impact of CL-containing membranes on the activity was tested with small unilamellar vesicles (SUVs), formed from a mixture of phosphatidylcholine (PC) and CL, which mimic mitochondrial membranes, activating the peroxidase activity of C*c* [33,34,35,36].

The assay mixtures were incubated for 24 h and at the end the reaction media was extracted with hexane for HPLC analysis. Two extraction procedures were employed, essentially differing in the number of extraction runs, as described in Section 3.5. The procedure with two extraction runs was optimized in our laboratory for the complete recovery of different PAHs with three to five benzene rings. In the present work, the efficiency of this procedure was assessed with the smaller and less hydrophobic (log K_o/w_ < 5) compounds studied, naphthalene, acenaphthene, anthracene, and pyrene; and the two HO-PAH metabolites. The results showed that the recovery was superior to 94% for all those compounds, except for 2-hydroxynaphthalene. For this metabolite (log K_o/w_ = 2.7), it was necessary to extend the extraction procedure to four runs, achieving a higher efficiency of 80 ± 8%. For the more hydrophobic and less water-soluble PAHs (log K_o/w_ > 5, see Table S1), an efficiency of extraction close to 100% was expected and the shorter two-runs procedure was followed.

### 2.1. Transformation of Polycyclic Aromatic Hydrocarbons by Cytochrome c

The initial set of assays focused on three PAHs—naphthalene (2 rings), pyrene (4 rings), and BaP (5 rings)—encompassing wide differences in the molecular weight and ionization potential (Table S1). In addition to reaction mixtures with C*c* alone (in the absence of phospholipids), parallel assays were carried out with C*c* in the presence of phospholipid SUVs: vesicles composed only of PC, and others of PC and CL in a 4:1 ratio (Section 3.2).

**Figure 1:**
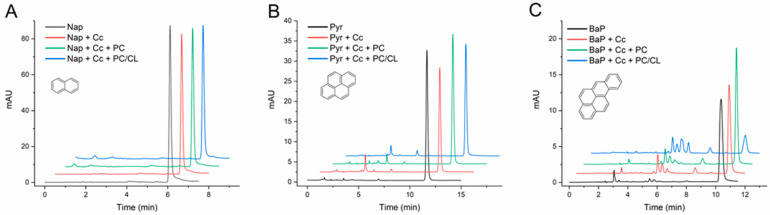
HPLC chromatograms of naphthalene (**A**), pyrene (**B**), and benzo[a]pyrene (**C**) transformation assays by cytochrome *c* (C*c*) in the absence and in the presence of PC and PC/CL small unilamellar vesicles. Chromatograms are shown for controls of each compound (1 mg/L) incubated in the absence of C*c*, of the compound with C*c* 0.01 mg/mL, and with and without PC and PC/CL vesicles (200 µM). All assay media included 100 µM H_2_O_2_ in 20 mM phosphate buffer pH 7.0 and were incubated for 24 h at 37 °C. The chromatograms are displaced in the vertical and horizontal axes for better observation. The chromatograms shown are representative results of triplicate assays for each reaction condition. The molecular structures of the compounds are depicted.

The results in Figure 1 and Table 1 show that C*c* was unable to catalyze any significant transformation of naphthalene in the conditions of the assays, but it catalyzed the conversion of pyrene and BaP into two or more products each. In the case of naphthalene, small decreases in the chromatographic peak of the PAH were measured from the assays containing C*c* relative to the controls without protein, as presented in Figure 1A, but the differences were not significant (Table 1). Furthermore, the chromatograms from these assays did not reveal a consistent generation of any product of the reaction. Yet, as observed in the chromatograms (see at tR approx. 2 and 5 min, Figure 1A), small peaks were occasionally present in the analysis of assay media containing C*c* and naphthalene, but they were not constant throughout the study.

On the contrary, C*c* catalyzed the transformation of pyrene and BaP with the generation of reaction products clearly distinguishable in the chromatograms (Figure 1B,C). When PC vesicles were present in the reaction media, the conversion of these PAHs was not substantially affected, but the SUVs containing CL greatly increased the C*c*-catalyzed transformation of BaP (Figure 1C and Table 1). In the presence of the PC/CL vesicles, the conversion of BaP reached almost 70% and gave rise to six detectable reaction products (see tR from 5 to 8 min in Figure 1C).

The quantification of the degree of transformation, as presented in Table 1, revealed the efficiency of C*c* in the conversion of BaP in the presence of CL membranes. Several additional control assays were carried out with BaP to rule out PAH transformation by some non-catalyzed reaction with H_2_O_2_ and/or the lipids. As presented in Table S2, the BaP measured in the samples after incubations showed no significant alterations, and the chromatograms did not reveal the formation of any reaction products. These results clarify that the enzyme-independent oxidation of BaP by H_2_O_2_ is negligible, even in the presence of the PC/CL vesicles. In addition, the hypothesis that PC/CL lipids could react with BaP in the presence of C*c* by some mechanism cumulative to H_2_O_2_ oxidation was discarded.

The differential effect of the PC and PC/CL membranes on the peroxidase activity of C*c* is in line with previous data on the C*c*-catalyzed transformation of methyl orange [38] and prototypical peroxidase substrates [34,35,36]. While C*c* does not interact specifically with PC, and the peroxidase activity is thus not altered, the binding to CL triggers alterations in the conformation of the polypeptide chain that reflect in the redox reactivity of the heme group and favor the peroxidase activity of C*c* [25,33,34,36].

It should be noted that CL-induced activation of the peroxidase activity of C*c* has been shown with relatively polar substrates, namely 2,2′-azino-bis-(3-ethylbenzothiazoline-6-sulphonate (ABTS), Amplex Red, guaiacol, and etoposide [25,34,35,36], which are all chemical species considerably different from the highly hydrophobic PAHs now studied.

Back to the PC membranes, a plausible effect of their presence in the reaction media is to sequester the lipophilic PAHs and limit their availability to reaction with C*c* in solution. Although this effect is out of the interest of the present work, it explains why the conversion of pyrene eventually seems to decrease in the presence of these lipid membranes (Table 1). In any case, there was no relevant reason to continue experiments with the PC vesicles.

**Figure 2:**
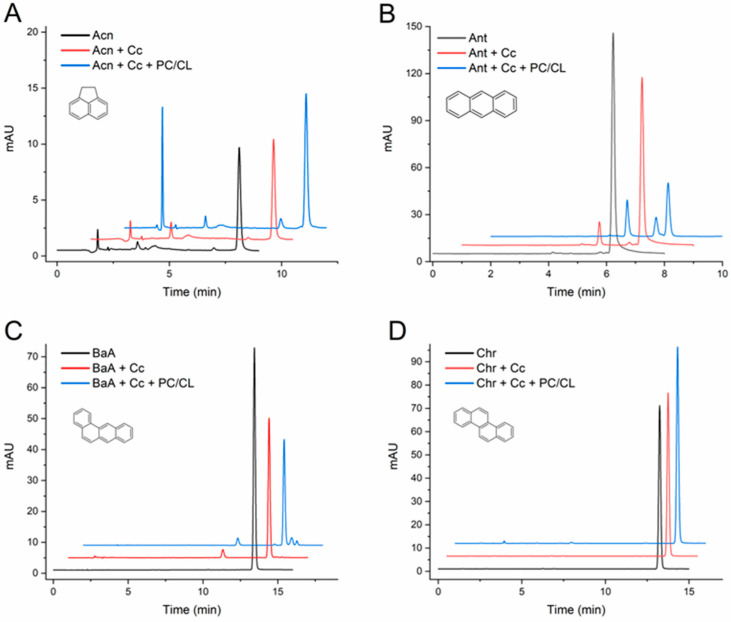
HPLC chromatograms of acenaphthene (**A**), anthracene (**B**), benzo[a]anthracene (**C**), and chrysene (**D**) transformation assays by cytochrome *c* (C*c*) in the absence and in the presence of PC/CL small unilamellar vesicles. Chromatograms are shown for controls of each compound (1 mg/L) incubated in the absence of C*c*, the compound with C*c* 0.01 mg/mL, and with and without PC/CL vesicles (200 µM). All assay media included 100 µM H_2_O_2_ in 20 mM phosphate buffer pH 7.0 and were incubated for 24h at 37 °C. The chromatograms are displaced in the vertical and horizontal axes for better observation. The chromatograms shown are representative results of triplicate assays for each reaction condition. The molecular structures of the compounds are depicted.

At this point, it was important to extend the study and evaluate the effect of the CL-containing membranes with a larger series of PAHs. Table 1 summarizes the quantification of the changes observed in the assays with five more PAHs: acenaphthene, anthracene, BaA, chrysene, and BbF. Representative chromatograms from the assays are presented in Figure 2, and for BbF in the abstract published before [38]. The results clearly indicate that C*c* was unable to transform chrysene, whereas it catalyzed the conversion of the other PAHs into two or more products each. Moreover, the vesicles made of a mixture of PC and CL enhanced the C*c*-mediated transformations, as evidenced by the decrease in the chromatographic peaks of the original PAH and/or by the notable emergence of reaction product peaks repeatedly observed in the chromatograms.

Regarding the products, it is worth commenting that low-molecular-weight acenaphthene gave rise to a prominent peak with very low tR, indicative of a highly hydrophilic product nearly not retained by the C18 column, in addition to two other peaks with longer tRs (Figure 2A). Except in the case of BaA (Figure 2C), all the detected reaction products of the PAHs had a shorter tR than the parent compounds, following the probable hypothesis that they are quinone and hydroxylated forms of the PAHs.

It is reasonable to assume that C*c* reacts with the PAHs as described for other heme peroxidases like CYP450, HRP, and prostaglandin H synthase (PGHS, or cyclooxygenase). In these enzymes, the PAHs transfer one electron to the peroxide-activated enzyme with Fe in a highly oxidized form [24,25,29,37,39]. The produced PAH radical cations are reactive electrophilic species that can rapidly undergo further oxidations by oxygen to form quinones and hydroxylated PAHs, but in biological systems they can also bind to DNA and form DNA adducts [37,39].

In the case of pyrene, the product detected with a tR of 5 min (Figure 1B) is probably a mono-hydroxylated form of pyrene, since 1-hydroxypyrene was observed in our analyses with the same tR as that presented in Section 2.3. However, the dynamics and identification of the products and intermediates generated in the reaction systems are out of the scope of the present work and require further analytical studies.

Overall, the assays presented in this section add novel and significant data about the ability of C*c* to catalyze the transformation of PAHs and how it is amplified in the presence of CL membranes. A previous study reported that C*c* catalyzed the H_2_O_2_-triggered oxidation of anthracene, pyrene, and BaP, but not of acenaphthene, chrysene, naphthalene, or other PAHs with very high ionization potentials, indicating their strong resistance to oxidation [28]. Our results generally confirm the previous report, except for concerning acenaphthene. It is worth noting that our assays at pH 7.0 also indicate the low activity of C*c* towards acenaphthene, and the demonstration of the actual reaction is supported by the appearance of reaction products (and the results at pH 5.0 presented in the next section) so it could easily go unnoticed without a full examination of the chromatographic results. More significantly, the present work demonstrates that C*c* can catalyze the transformation of several important PAHs, including those not previously studied, BaA and BbF, under less-favorable yet closer-to-physiological conditions, namely with H_2_O_2_ at sub-millimolar levels and at pH 7.0.

Moreover, the degree of C*c*-mediated conversions of PAHs of major toxicological relevance, such as BaP, anthracene, BaA, and BbF, are even more extended in the presence of phospholipid vesicles analogous to cell mitochondrial membranes. When compared to other peroxidases and CYP450, C*c* was illustrated to have a low catalytic activity for PAH oxidation [29]. However, the present data at neutral pH and in the presence of CL-containing membranes indicate that C*c* and C*c*-CL complexes might reach oxidizing potencies towards critical PAHs similar to or higher than common peroxidases.

### 2.2. Comparison of Cytochrome c with Horseradish Peroxidase and Determination of the Ionization Potential Threshold of Cytochrome c

The ability of peroxidases to oxidize PAHs has been correlated with the ionization potentials of the compounds, i.e., the energy necessary to remove an electron from the neutral PAH molecule to generate a cation radical [23,24,37,39]. The prototypical plant peroxidase HRP is accepted as able to oxidize PAHs with an ionization potential of up to 7.2 eV (BaP), but it is not effective towards anthracene (7.4 eV), nor pyrene (≥7.5 eV) or sturdier compounds [37]. An ionization potential threshold of 7.35 eV was assumed for HRP, as well as for PGHS [39], which are inferior to some other peroxidases [23], but there is no such estimation for the peroxidase forms of C*c*.

Table S1 lists the ionization potentials of the PAHs studied herein [40]. Naphthalene and phenanthrene are common PAHs with very high ionization potentials (≥8.0 eV), making these compounds very resistant to biodegradation [24]. So, it is not surprising that neither HRP [37] nor the peroxidase activity of C*c* are sufficiently potent to oxidize naphthalene, even in the presence of CL membranes (Figure 1A). On the same basis, the ability of both HRP [37] and C*c* to transform BaP (Figure 1C) can be justified by the relatively low ionization potential of this PAH (Table S1). However, C*c* in the free form and, especially, when complexed to CL transformed anthracene and BaA in sizable extensions (Figure 2B,C). These results suggest that C*c* has an ionization potential threshold superior to HRP, which did not oxidize anthracene, pyrene, or BaA [37]. Reinforcing that hypothesis, the E^0^[Fe(III)/Fe(II)] of approximately −0.17 V reported for C*c*-CL complexes [33] is superior to the values close to −0.3 V attributed to HRP [24]. Still, some doubts could be raised since our results with acenaphthene at pH 7.0 are contradictory to those previously reported for C*c* [28]; furthermore, acenaphthene and chrysene have not been tested before with HRP [37].

Therefore, a set of transformation assays were performed to further assess the ability of HRP and C*c* to oxidize PAHs with intermediate-to-high ionization potentials: BaP, anthracene, pyrene, acenaphthene, and chrysene (values from 7.17 to 7.74 eV in Table S1). These assays were carried out at pH 5.0, a condition known to promote peroxidase activity [25,26], and with a higher concentration of catalyst to favor extended and clearly recognizable transformations.

**Figure 3:**
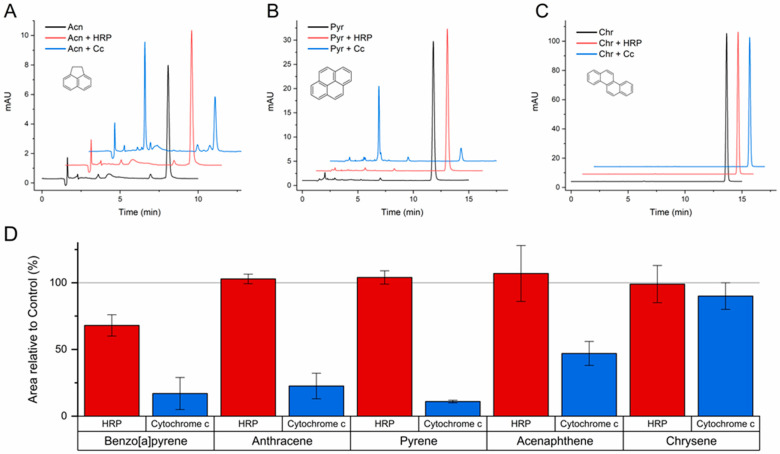
Transformation of acenaphthene (**A**), pyrene (**B**), chrysene (**C**), and other PAHs (**D**) catalyzed by horseradish peroxidase (HRP) and cytochrome *c* (C*c*) at pH 5.0. The reaction media contained the PAHs at an initial concentration of 1 mg/L in 100 mM acetate buffer, HRP at 0.2 µg/mL or C*c* at 0.1 mg/mL, and H_2_O_2_ at 100 μM. After 24 h incubations at 37 °C, the reaction media was analyzed by HPLC. The area of the chromatographic peak corresponding to the remaining parent compound was compared to the results from parallel control assays (no protein), which were taken as 100%. The mean ± SE of the data from triplicate assays are represented in panel D for the PAHs tested and ordered by increasing ionization potential. The chromatograms shown in (**A**–**C**) are representative results of triplicate assays for each compound (molecular structures depicted) and each catalyst.

The results confirmed the ability of both HRP and C*c* to catalyze the reaction of BaP with H_2_O_2_, and also that only C*c* can transform anthracene and pyrene. As shown in Figure 3A,B, acenaphthene and pyrene were substantially transformed by C*c* in the conditions of these assays. However, none of the catalysts showed enough oxidizing potency to attack chrysene (Figure 3C), despite the favorable pH and concentrations used. The conversion degrees quantified for each catalyst are represented in Figure 3D, showing the compounds ordered by increasing ionization potential. In addition to the confirmation of the cut-off of HRP between BaP and anthracene, the results of C*c* exhibit quite a good correlation with the ionization potentials, and a sharp threshold can be determined. Since C*c* was able to oxidize PAHs with ionization potentials up to 7.70 eV (acenaphthene), but not with 7.74 eV (chrysene), an ionization potential threshold of 7.70 eV can be concluded for the peroxidase activity of C*c*. This oxidizing potency is more characteristic of fungal ligninolytic peroxidases and less of plant peroxidases like HRP [23,24,29], implying the possibility of using C*c* for novel industrial and bioremediation applications.

### 2.3. Transformation of Polycyclic Aromatic Hydrocarbons Metabolites by Cytochrome c

Facing the capacity of C*c* to modify PAHs, we decided to investigate if it could also transform their hydroxylated forms, which are well known in vivo metabolites and biomarkers of PAH exposure. Two HO-PAHs were selected for study, 2-hydroxynaphthalene and 1-hydroxypyrene, one low- and one high-molecular-weight metabolite that are frequently measured in greater levels in exposed individuals [2,5,10] and associated with pathological alterations [6,7,10,11].

**Figure 4:**
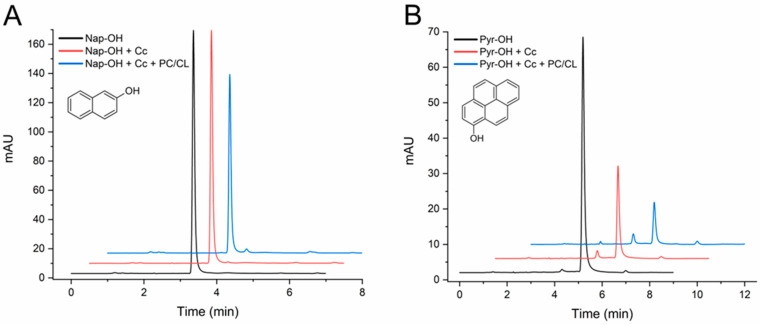
HPLC chromatograms of 2-hydroxynaphthalene (**A**) and 1-hydroxypyrene (**B**) transformation assays by cytochrome *c* (C*c*) in the absence and in the presence of PC/CL small unilamellar vesicles. Chromatograms are shown for controls of each compound (1 mg/L) incubated in the absence of C*c*, the compound with C*c* 0.01 mg/mL, and with and without PC/CL vesicles (200 µM). All assay media included 100 µM H_2_O_2_ in 20 mM phosphate buffer pH 7.0 and were incubated for 4 h at 37 °C. The chromatograms are displaced in the vertical and horizontal axes for better observation. The chromatograms shown are representative results of triplicate assays for each reaction condition. The molecular structures of the compounds are depicted.

The initial assays carried out with 24 h incubations proved that C*c* catalyzed the transformation of both HO-PAHs. However, as shown in Figure S1 of the Supplementary Material, extensive conversions occurred, even in the absence of SUVs, so the effect of CL could not be clearly determined. A different group of assays was then carried out by incubations of 4 h (Figure 4). With this shorter time, the conversion of 2-hydroxynaphthalene was small, approximately 10%, but in the presence of the CL-containing vesicles it reached 24% (Table 1). The results at 4 and 24 h show that 1-hydroxypyrene is more efficiently converted than 2-hydroxynaphthalene (Figure 4B and Figure S1B). Also, with 1-hydroxypyrene, the CL membranes increased the conversion degree from 59% to 80% (Table 1).

As mentioned before, 1-hydroxypyrene was observed in the analyses with a tR of 5 min, and a reaction product was detected approximately 1 min before (Figure 4B). These tRs coincide with two of the products generated in the assays with pyrene (Figure 1B), suggesting that some initial products of PAH oxidation can be further transformed by C*c*.

In the mitochondria of healthy cells, it is assumed that most C*c* is free and only a minor fraction is tightly bound to the inner mitochondrial membrane [41]. However, events that alter the distribution of CL in the mitochondrial membranes can vary the proportion of free to membrane-bound C*c*, and during apoptosis quantities of C*c* are released to the cytosol. It should be noted that small pools of C*c* also appear in the cytosol of non-apoptotic cells [31]. Moreover, C*c* can be present in the extracellular milieu, and elevated levels of circulating C*c* were detected in conditions associated with mitochondrial damage like heart and liver disease [41]. Therefore, considering the novel data presented in this work, free and lipid-associated C*c* can play an important role in the metabolism of PAHs and HO-PAHs, especially in the mitochondria, but also in the cytosol and outside the cells in specific conditions. The relatively high ionization potential cut-off and efficiency towards dangerous PAHs like BaP, anthracene, and BaA indicate that C*c* can contribute to the generation of reactive metabolites and to the toxicity of PAHs in mitochondria [14,15,16,17].

### 2.4. Spectral Studies of Cytochrome c in the Presence of Polycyclic Aromatic Hydrocarbons

It is well accepted that C*c* reacts with small molecules in solution by outer-sphere electron-transfer processes via the exposed heme edge, although alternative mechanisms of redox reaction involving binding to the protein and/or different electron migration pathways have been discussed [31]. It is also recognized that C*c* has binding sites with significant affinity for lipids and amphiphilic molecules [34,35,36], so it is questionable as to whether the PAHs directly interact with C*c*.

**Figure 5:**
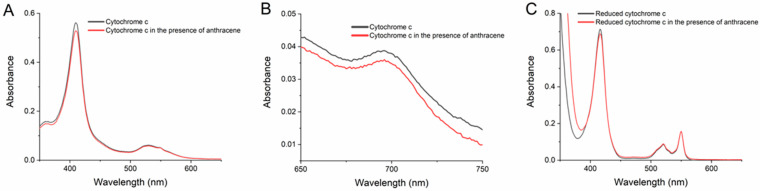
Spectra of cytochrome *c* (C*c*) in the UV-visible region (**A**,**C**) and in the near-infrared region (**B**) in the absence and in the presence of anthracene. The concentration of C*c* was 5 µM in panels (**A**,**C**), and 50 µM in (**B**) in 20 mM phosphate buffer pH 7.0. Anthracene was added to the C*c* solutions reaching equimolar concentrations, and spectra were collected after 2 min of incubation. In panel (**C**), C*c* in the absence and in the presence of anthracene was reduced with dithionite, as indicated in the Methods.

This hypothesis was tested by examining the effect of the PAHs in the absorption spectra of C*c* in solution in the same buffered media at pH 7.0 of the transformation assays presented in Section 2.1 and Section 2.3. The spectra of C*c* (ferriC*c*) exhibited the characteristic heme absorption bands, namely the Soret band with a peak at 409 (or 410) nm in the UV-Vis region (Figure 5A) and the charge-transfer (CT) band in the near-infrared at 695 nm (Figure 5B). All the PAHs and HO-PAHs were tested, but anthracene was the only one that consistently induced a greater change in the C*c* spectra. Representative UV-Vis spectra of C*c* with and without anthracene are given in Figure 5A. Spectra from the studies with the remaining nine compounds are presented in Figure S2. Additional assays of prolonged incubations of C*c* with anthracene discarded the hypothesis that the decrease in the Soret band augmented with time.

The CT band is lost when C*c* is reduced to ferroC*c* or when ligands disturb the coordination of the heme Fe to the methionine-80 residue in the polypeptide chain [34,36]. As shown in Figure 5B, anthracene caused no significant changes in the shape or intensity of this band. The reduction in C*c* also provokes a red shift of the Soret band and the appearance of 520 and 550 nm bands [33,36], as observed in Figure 5C for C*c* reduced with dithionite (ferroC*c*). However, the spectra of ferriC*c* incubated with anthracene (Figure 5A) or with the other compounds (Figure S2) did not give any sign of such alterations, not even with the HO-PAHs. Instead, the Soret band of ferroC*c* showed a slight decay after incubation with anthracene (Figure 5C), like the one observed with ferriC*c* (Figure 5A).

In this context, it is relevant to notice that, in a previous study, dibenzothiophene interaction with C*c* also caused a small decrease in the Soret band of the protein, which was registered in the absence of H_2_O_2_ to avoid any possible redox reaction [27]. In this work with several organosulfur compounds, H_2_O_2_ oxidation of dibenzothiophene was efficiently catalyzed by C*c*. Remarkably, our transformation assays and a previous work [28] point out that C*c* has a relatively high catalytic activity with anthracene.

The present results from the spectral studies indicate that the PAHs do not cause large alterations in the structure of C*c*. However, at least anthracene binds to C*c* in a way that slightly affects the heme environment and, thus, might contribute to the catalytic mechanism.

### 2.5. Prediction of Anthracene Docking to Cytochrome c

Taking into account the results from the spectral studies with anthraceneand that this important prototypical PAH was efficiently transformed by C*c* (Figure 2B), the binding of anthracene was investigated by docking simulations.

**Figure 6:**
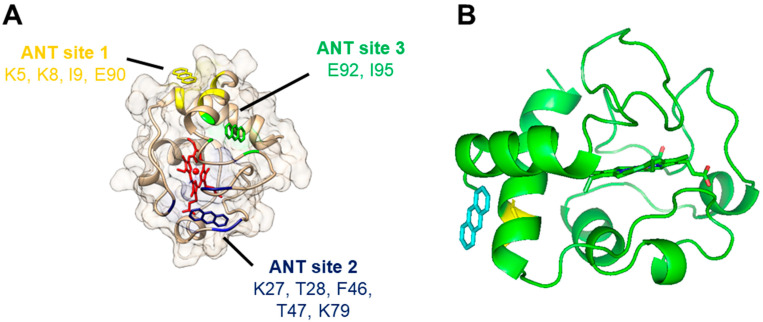
Docking of anthracene to cytochrome *c* (PDB 1HRC) as predicted by molecular simulations: (**A**) General view of the three predicted binding sites of anthracene to cytochrome *c*. Cytochrome *c* backbone and surface are shown in light brown, and the heme group in red. Anthracene molecules interacting with its modeled binding sites 1, 2, and 3 are shown in yellow, dark blue, and green, respectively. (**B**) Detail of the first pose of the top-ranked models of anthracene docked at cytochrome *c* (site 1). The location of an anthracene molecule (labeled in blue) interacting with cytochrome *c* (backbone and heme in green) is depicted in relation to the location of cytochrome c’s Glu90 residue (labeled in yellow).

The molecular docking to horse heart C*c* was computed with AutodockVina to predict models for the interaction of anthracene (Table 2 and Figure 6). The results pointed out that anthracene can interact with C*c* in different locations (Table 2). Our docking analysis suggests three possible binding sites with very similar probabilities based on the binding energy (Table 2). Anthracene was able to bind to site 1, defined by amino acid residues Lys5, Lys8, Ile9, and Glu90, with a binding energy of −5.2 kcal/mol. We also found that it was able to bind to site 2, defined by amino acid residues Lys27, Thr28, Phe46, Thr47, and Lys79, with a binding energy of −5.0 kcal/mol, and site 3, defined by amino acid residues Glu92 and Ile95, with a binding energy of −5.4 kcal/mol.

As depicted in Figure 6A, anthracene does not interact directly with the heme group, but the three predicted binding sites are close to or in regions connected to the heme. Therefore, the binding of anthracene might eventually induce a small alteration in the conformation of C*c* sufficient to affect the heme environment, as suggested by the spectral studies (Figure 5A).

It should be noted that some of the residues pointed to interact with anthracene (Table 2) have been implicated in important biological functions of C*c*. Lys27 is part of the so-called L-binding site of CL, and the residues 5, 8, and 9 are in very close proximity to this CL binding site [42]. Similarly, Lys27 is one of the several lysine residues involved in C*c* interaction with apoptotic protease-activating factor-1 (Apaf-1) in the formation of the apoptosome [43]. However, there are other binding sites for CL under debate, and the L site includes more than 15 residues [42], while Apaf-1 binding also involves other residues like Lys72, which is more accepted as critical [43]. Therefore, a significant interference of anthracene with these C*c* functions seems unlikely.

Site 1 for anthracene captured our attention since several high-ranked poses at this site were returned from the (triplicate) docking experiments and, in another study, Glu90 was directly involved in the binding of imidazole-substituted fatty acids that inhibit the peroxidase activity of C*c* complexes [35]. The molecular docking of these inhibitors proposed their binding to the Glu90 region at the entrance of a hydrophobic channel, directing the molecules to the proximity of the heme group. The top-ranked pose of anthracene at site 1 is shown in Figure 6B.

However, Lys79 (site 2) has also been implicated in the binding of C*c* redox partners, both proteins and small molecules, for the efficient electron transfer from or to C*c* [31,44], which eventually occurs through the Tyr48 located very close to the heme group [45]. It is tempting to speculate that PAH binding to site 1 or 2 disposes the molecules for electron subtraction by C*c*, but more molecular details are needed in the future to fully understand the redox reactions of PAHs and C*c*.

## 3. Materials and Methods

### 3.1. Chemicals 

The commercial suppliers of the PAH reagents used, along with the respective catalog number and purity, are listed in Table S1 of the Supplementary Material. The stock solutions of the PAHs were prepared at concentrations of 100 or 200 mg/L in acetonitrile. 

The C*c* (from equine heart, cat. no. C2506) was obtained from Sigma-Aldrich, Algés, Portugal, as well as HRP (cat. no. P6782). Diethylenetriaminepentaacetic acid (DTPA) was from Merck, Darmstadt, Germany (cat. no. D6518, purity ≥ 99%) and hexane from Fisher Chemical, Geel, Belgium (H/0355/17, ≥99%); PC was from Tokyo Chemical Industry, Tokyo, Japan (D4250) and tetraoleoyl CL from Avanti, Alabaster, AL, USA (710335P). All other chemicals used were HPLC or analytical-grade reagents.

### 3.2. Phospolipid Membranes

SUVs composed of PC or mixtures of PC with CL at a 4:1 molar ratio were obtained as described in [36]. Briefly, chloroform solutions of the phospholipids were dried and resuspended in 20 mM sodium phosphate buffer (pH 7.0, supplemented with 100 μM DTPA) to a final concentration of 10 mM. Just before the catalysis assays, SUVs were prepared by sonication of 200 μL aliquots (in ice) of the phospholipid suspension, employing a titanium-microtip-equipped Hielscher UP100H sonicator until clarity of the suspension was achieved.

### 3.3. Cytochrome c Solutions and Spectral Studies

Stock solutions of C*c* were routinely prepared in distilled water and, just before the assays, diluted in the corresponding buffer. The concentration in the stock solutions was quantified after reduction with sodium dithionite and by using the extinction coefficient ε(550 nm) = 27.6 mM^−1^ cm^−1^.

The absorption spectra of C*c* in 20 mM sodium phosphate buffer (pH 7.0) were collected using a Varian Cary 50 UV-Vis spectrophotometer. The protein concentration was 5 or 50 μM, as indicated in the figure captions. The effect of the equimolar concentration of each PAH was assessed by the direct addition of small volumes to the C*c* solution and collection of the spectra approximately 2 min after gentle homogenization of the mixtures. The absorbance of the compounds registered in the same wavelength range was subtracted from the spectra of the mixtures. For the spectra of reduced C*c*, the addition of a small amount of sodium dithionite was enough to reduce the protein heme in a few seconds, as is performed in a common procedure [33,36].

### 3.4. Peroxidase-Catalyzed Reaction Assays

For the catalytic assays of C*c* at pH 7.0, the PAHs and metabolites were diluted in 20 mM sodium phosphate buffer (pH 7.0, supplemented with 100 μM DTPA) to a concentration of 1 mg/L. Depending on the compound, this mass concentration corresponds to between 4 and 8 μM. Since the stock solutions of the compounds were in acetonitrile, the reaction media contained up to 1% (*v*/*v*) of this solvent. C*c* was added at a concentration of 0.0125 mg/mL (1 μM), and H_2_O_2_ at 100 μM. Immediately after, the reaction tubes were tightly sealed and incubated in the absence of light at 37 °C for 24 h. Incubations of 4 h with the HO-PAHs were also carried out. In the assays with lipid membranes, the SUVs were added in a concentration of 200 µM for the total volume of the reaction media, before the addition of C*c* and H_2_O_2_.

The studies with C*c* and HRP at pH 5.0 followed the same procedure as above, except for that the reaction media was 100 mM sodium acetate buffer, and the protein concentrations were C*c* 0.1 mg/mL and HRP 0.2 µg/mL. These concentrations corresponded to 0.9 and 93 mU of peroxidase activity/mL (ABTS substrate), respectively.

In all sets of the assays, control tubes without the addition of any of the proteins were run in parallel to account for the actual PAH concentration in the reaction media.

### 3.5. HPLC Analysis

At the end of the incubations, hexane extracts of the reaction media were prepared for analysis by reverse-phase HPLC. Two extraction procedures were employed, differing in the number of hexane extraction runs. For the shorter method, a total of 1 mL of hexane was added in two 500 µL runs to 1 mL of reaction media, with 30 s of vigorous stirring in each run (protected from light). The two organic phases were collected after each run and combined for posterior HPLC analysis. This procedure was followed in the assays of all the compounds, except for 2-hydroxynaphthalene. For this less hydrophobic HO-PAH, an extended procedure with 4 extraction runs was employed. Starting with the 1 mL of reaction media, a first volume of 400 µL of hexane was added, followed by 1 min vigorous stirring and 1 min resting before the organic phase was collected. Two more 400 µL runs and one 300 µL run were carried out, and the combined 1.5 mL hexane extract was subsequently analyzed. The extraction efficiency of these procedures was assessed by measuring the recovery of the compounds from standards in phosphate buffer extracted and analyzed by HPLC as applied to the assay samples.

The HPLC analysis of the extracts was carried out in an Agilent 1100 system equipped with a C18 column by using a mobile phase of acetonitrile and water (85:15) at a 1 mL/min flux [46]. Two C18 columns were used in different periods of the work, a Zorbax Eclipse Plus (Santa Clara, CA, USA) and a Knauer Eurospher II (Berlin, Germany). The UV detection wavelengths were 220 nm for naphthalene; 251 nm for anthracene; 240 nm for pyrene; 266 nm for BaP and chrysene; 288 nm for BaA; 256 nm for BbF; 224 nm for acenaphthene and 2-hydroxynaphthalene; and 242 nm for 1-hydroxypyrene. The linearity of the response signal in the range of PAH concentrations measured in the assays was assessed based on the calibration curves obtained with standards of each of the compounds in hexane.

### 3.6. Molecular Docking of Anthracene to Cytochrome c

For the simulations, the file with an sdf extension containing the molecular structure of anthracene was downloaded from Zinc12 (ZINC01586329) [47] and transformed into files with extension mol2 using Openbabel 2.3.1 [48]. This tool was also used to minimize the structure of the ligand by applying the steepest descent algorithm, MMFF94 force field, and 50,000 steps for fitting after adding hydrogens to the molecule. We used Autodock Tools 4 (ver. 1.5.6) [49] to prepare the molecule as ligands for docking analysis. We visualized and set the torsion parameters, and then saved the file with the extension pdbqt for its submission for docking analysis. The molecular structure of C*c* was downloaded from the Protein Data bank (PDB file: 1HRC), which corresponds to the high-resolution three-dimensional structure of horse heart C*c*. For docking preparation, water molecules were removed from the structure, hydrogens were added (polar only), and Kollmand charges were added using Autodock Tools 4. The prepared file for docking was saved as a pdbqt file. The grid for the docking analysis was structured as follows: center x = 46.839, center y = 23.029, center z = 5.505 with a box size of: 52 for x, 52 for y and 52 for z.

The docking analysis was performed with AutodockVina 1.1.2 and the previously indicated files for the receptor (PDB:1HRC.pdbqt) and the ligand (anthracene.pdbqt) [50] by using the previously indicated grid for C*c* and an exhaustiveness value of 9. The top 10 ranked poses associated with models of the ligand–C*c* complex were visually analyzed using UCSF Chimera 1.15 [51] and clustered by homology. The binding site for anthracene was defined and selected based on the residues making contact and clashing with the ligand within a distance of 0.1 Å. We gathered similar poses together in subclusters for each experiment. Each experiment was performed in triplicate. A cluster was defined as the set of residues present in subclusters in all the performed individual experiments. The lowest energy value of the top-ranked pose present in a subcluster forming a cluster was selected as the energy value for the selected cluster.

## 4. Conclusions

PAHs are metabolized by three major pathways that generate reactive intermediates able to cause DNA mutations and oxidative damage to other biomolecules: (1) the CYP450/epoxide hydrolase pathway that produces epoxides; (2) aldo–keto reductases that lead to the formation of redox cycling PAH o-quinones; and (3) peroxidases that produce PAH radical cations which, in spite of being short-lived, can react with DNA, RNA, proteins, and lipids [8,9,21]. In addition to CYP450 peroxidase activity, other peroxidases like PGHS were implicated in the one-electron oxidation of PAHs to produce radical cations that react with DNA and cause mutations [21,37,39].

The results in this work indicate that C*c* is another possible source of PAH radical cations, which has special implications for the mitochondrial toxicity of PAHs. With moderate levels of H_2_O_2_ and at pH 7.0, C*c* catalyzes the oxidation of toxic PAHs, such as BaP, anthracene, BaA, and BbF, and does so more efficiently in the presence of CL-containing membranes. Future research should evaluate the genotoxic potential of the oxidized PAHs produced by C*c*. Also demonstrated for the first time, C*c* and C*c*-CL complexes were able to transform important HO-PAHs metabolites, suggesting that C*c* might participate in different steps of the metabolism of PAHs.

In a direct comparison with the canonical peroxidase HRP, C*c* was shown to reach more potent oxidizing states and react with PAHs with intermediate ionization potential, namely pyrene and acenaphthene, which are not attacked by HRP. The PAHs with an ionization potential of up to 7.70 eV were transformed by C*c*, and more extensively in the presence of CL membranes or at pH 5.0. However, chrysene (7.74 eV) was not oxidized, even in the presence of CL; therefore, the ionization potential threshold of the peroxidase activity of C*c* is 7.70 eV.

Spectral studies indicate that, at least, anthracene binds to C*c*, but none of the 10 PAHs/HO-PAHs studied were found to cause substantial alterations in the protein structure. The docking simulations proposed three possible binding sites of anthracene to C*c,* requiring further investigation to provide a full comprehension of the molecular interactions between PAHs and C*c*.

## Figures and Tables

**Table 1:** Summary of the transformation of polycyclic aromatic hydrocarbons and metabolites catalyzed by cytochrome *c* (C*c*) in the absence and presence of phospholipid membranes (PC-phosphatidylcholine; PC/CL- phosphatidylcholine and cardiolipin 4:1 mixture). The reaction media contained the PAHs at an initial concentration of 1 mg/L in 20 mM phosphate buffer (pH 7.0), C*c* at 0.01 mg/mL, and H_2_O_2_ at 100 μM. When lipid membranes (small unilamellar vesicles) were present, the total phospholipid concentration was 200 μM. After 24 h incubations (or 4 h when indicated) at 37 °C, reaction media was analyzed by using HPLC. The area of the chromatographic peak corresponding to the remaining parent compound was compared to the results from parallel control assays (no C*c*), which were taken as 100%. The data presented are the mean ± SE from triplicate assays.

Compound	Lipid Membranes	Area Relative to Control (%)
Naphthalene	Absent	90 ± 9
	PC	82 ± 10
	PC/CL	91 ± 10
Pyrene	Absent	79 ± 4 ^(1)^
	PC	105 ± 5 ^(1)^
	PC/CL	89 ± 3 ^(1)^
Benzo[a]pyrene	Absent	105 ± 13 ^(1)^
	PC	99 ± 16 ^(1)^
	PC/CL	29 ± 13
Acenaphthene	Absent	93 ± 12 ^(1)^
	PC/CL	155 ± 22 ^(1)^
Anthracene	Absent	79 ± 21 ^(1)^
	PC/CL	39 ± 14
Benzo[a]anthracene	Absent	65 ± 7
	PC/CL	48 ± 4
Chrysene	Absent	91 ± 12
	PC/CL	109 ± 9
Benzo[b]fluoranthene	Absent	91 ± 4
	PC/CL	79 ± 6 ^(1)^
2-Hydroxynaphthalene (t = 4 h)	Absent	87 ± 9 ^(1)^
	PC/CL	76 ± 12
1-Hydroxypyrene	Absent	41 ± 4
(t = 4 h)	PC/CL	20 ± 4

^(1)^ In spite of the small decreases in the peak area of the parent compound (variation < 20%), reaction products were constantly detected in the chromatograms, and/or larger conversions were evident with longer incubation times.

**Table 2:** Binding sites of anthracene to cytochrome *c* (PDB 1HRC) predicted by docking simulations.

Cluster	Binding Energy (kcal/mol)	Interacting Residues
Site 1 named as ANT 1	−5.2	Lys5, Lys8, Ile9, Glu90
Site 2 named as ANT 2	−5.0	Lys27, Thr28, Phe46, Thr47, Lys79
Site 3 named as ANT3	−5.4	Glu92, Ile95

## Data Availability

The data are presented within the article and available from the authors (R.L.) on request.

## Supplementary Materials

The following supporting information can be downloaded at: https://www.mdpi.com/article/10.3390/molecules29051129/s1, Table S1: Polycyclic aromatic hydrocarbons studied in this work; Table S2: Control assays of benzo[a]pyrene transformation in the absence of cytochrome *c* or in the absence of H_2_O_2_; Figure S1: HPLC chromatograms of 2-hydroxynaphthalene and 1-hydroxypyrene transformation assays (24 h); Figure S2: UV-Vis absorption spectrum of cytochrome *c* in the presence of naphthalene, acenaphthene, pyrene, benzo[a]anthracene, chrysene, benzo[a]pyrene, benzo[b]fluoranthene, 2-hydroxynaphthalene and 1-hydroxypyrene.
