# Supplementary material for: Cardiolipin Membranes Promote Cytochrome c Transformation of Polycyclic Aromatic Hydrocarbons and Their In Vivo Metabolites
Source: Molecules. 2024 Mar 3;29(5):1129. doi: 10.3390/molecules29051129 (PMC10935164; doi:10.3390/molecules29051129)
Supplement: Supplementary file 1 [file molecules-29-01129-s001.zip › molecules-2826302-supplementary.pdf]

Supplementary Material

Cardiolipin membranes promote cytochrome *c* transformation of polycyclic aromatic hydrocarbons and their *in vivo* metabolites

(Lopes *et al.*, *Molecules*, 2024)

Table S.1
Polycyclic aromatic hydrocarbons studied in this work.

Table S.2
Control assays of benzo[a]pyrene transformation in the absence of cytochrome *c* or in the absence of H_2O_2.

Figure S.1
HPLC chromatograms of 2-hydroxynaphthalene and 1-hydroxypyrene transformation assays (24 h).

Figure S.2
UV-Vis absorption spectrum of cytochrome *c* in the presence of naphthalene, acenaphthene, pyrene, benzo[a]anthracene, chrysene, benzo[a]pyrene, benzo[b]fluoranthene, 2-hydroxynaphthalene and 1-hydroxypyrene.

Table S.1. Polycyclic aromatic hydrocarbons studied in this work.

Compound	CAS number	Molecular weight (g mol^{-1})	log $K_{o/w}$ *	Water solubility (mg mL^{-1}) **	Ionization potential (eV) ***	Supplier, catalogue number and purity
Naphthalene	91-20-3	128.2	3.30	1.19×10^{-2}	8.14	Merk 820846, ≥ 99%
Acenaphthene	83-32-9	154.2	3.92	3.80×10^{-3}	7.70	Sigma-Aldrich 215376, 99%
Anthracene	120-12-7	178.2	4.44	3.20×10^{-4}	7.42	Sigma-Aldrich A89200, ≥ 97%
Pyrene	129-00-0	202.25	4.88	5.22×10^{-5}	7.56	ACROS Organics A0322434, 98%
Benzo[a]anthracene	56-55-3	228.3	5.76	8.46×10^{-6}	7.47	Tokyo Chemical Industry B0017, 98%
Chrysene	218-01-9	228.3	5.81	8.46×10^{-6}	7.74	Tokyo Chemical Industry C0339, > 98%
Benzo[a]pyrene	50-32-8	252.3	5.97	1.38×10^{-6}	7.17	Sigma-Aldrich B1760, ≥ 96%
Benzo[b]fluoranthene	205-99-2	252.3	5.78	1.38×10^{-6}	n/a	Tokyo Chemical Industry B2982, ≥ 98%
2-Hydroxynaphthalene	135-19-3	144.2	2.70	$4.84 \text{ x } 10^{-2}$	n/a	Tokyo Chemical Industry N0027, ≥ 99%
1-Hydroxypyrene	5315-79-7	218.25	4.64	$2.11 \text{ x } 10^{-4}$	n/a	Tokyo Chemical Industry H1435, ≥ 99%

* Logarithm of the octanol/water partition coefficient (log $K_{o/w}$) computed by the XLOGP3 method and retrieved at the SwissADME web tool (http://www.swissadme.ch/).

** Water solubilities calculated by FILTER-IT program and retrieved at the SwissADME web tool (http://www.swissadme.ch/).

*** First ionization potentials in Lee and Hites (1977) Mixed charge exchange-chemical ionization mass spectrometry of polycyclic aromatic hydrocarbons, *J. Am. Chem. Soc.* 99(6), 2008-2009, DOI: 10.1021/ja00448a070

n/a – not available.

Table S.2. Control assays of benzo[a]pyrene (BaP) transformation in the absence of cytochrome *c* (C*c*) or in the absence of H_2O_2. The effect of the presence of small unilamellar vesicles of phosphatidylcholine and cardiolipin (PC/CL, 4:1 molar ratio) was also tested. All assays were carried out with an initial concentration of BaP 1 mg/L in 20 mM phosphate buffer pH 7.0. Additional components were added to the assay media as indicated. After 24 h incubation at 37 ºC, the media was extracted with hexane and analyzed by HPLC. The area of the peak of BaP in each tested condition was compared to control assays with only BaP in buffer, which were taken as 100%. The data presented are the Mean ± SE from triplicate assays for each condition.

**Condition**	**H_2O_2 (µM)**	**Cytochrome *c* (µM)**	**PC/CL vesicles (µM)**	**Area of BaP peak (%)**
BaP only (control assays)	0	0	0	100 ± 11
Presence of H_2O_2, but no C*c*	100	0	0	102 ± 18
Copresence of H_2O_2 and CL, but no C*c*	100	0	200	85 ± 15
Copresence of CL and C*c*, but no H_2O_2	0	1	200	104 ± 15

Figure S.1. HPLC chromatograms of 2-hydroxynaphthalene (A) and 1-hydroxypyrene (B) transformation assays by cytochrome *c* (C*c*), in the absence and in the presence of PC/CL small unilamellar vesicles. Chromatograms are shown for controls of each compound (1 mg/L) incubated in the absence of C*c*, the compound with C*c* 0.01 mg/mL (1µM), without and with PC/CL vesicles (200 µM). All assay media included 100 µM H_2O_2 in 20 mM phosphate buffer pH 7.0, and were incubated for 24 h at 37 °C. The chromatograms are displaced in the vertical and horizontal axis for better observation. The chromatograms shown were obtained with a Zorbax Eclipse Plus C18 column and are representative results of triplicate assays for each reaction condition.

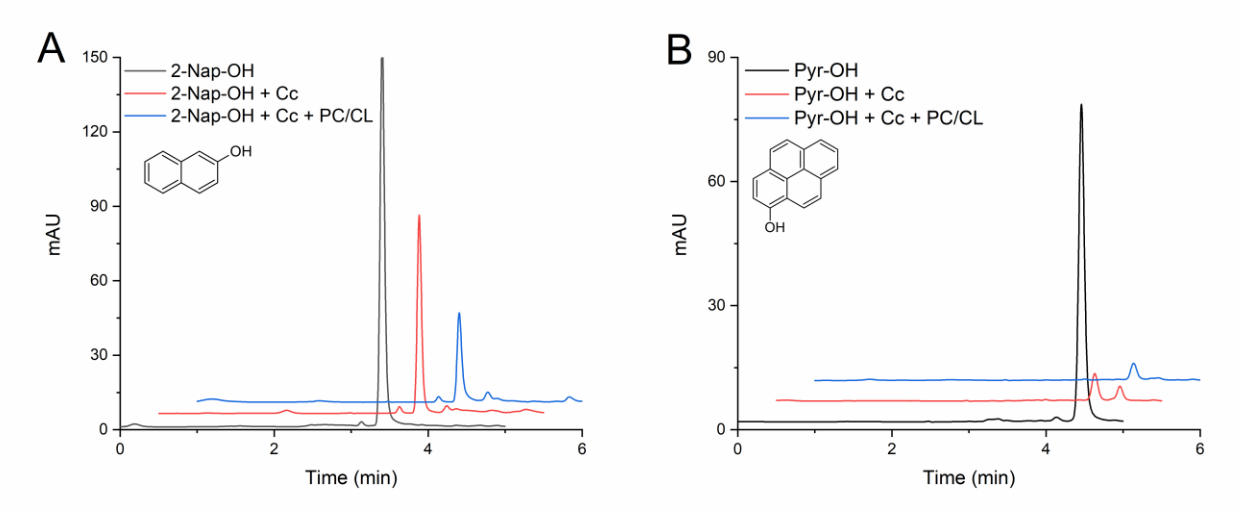

Figure S.2. UV-Vis absorption spectra of cytochrome *c* (C*c*) in the presence of naphthalene (A), acenaphthene (B), pyrene (C), benzo[a]anthracene (D), chrysene (E), benzo[a]pyrene (F), benzo[b]fluoranthene (G), 2-hydroxynaphthalene (H) and 1-hydroxypyrene (I). The concentration of C*c* was 5 µM, in 20 mM phosphate buffer pH 7.0. After recording the C*c* spectra, small volumes of the compound were added to the cuvette to reach equimolar concentration and the spectra were collected after 2 minutes incubation.

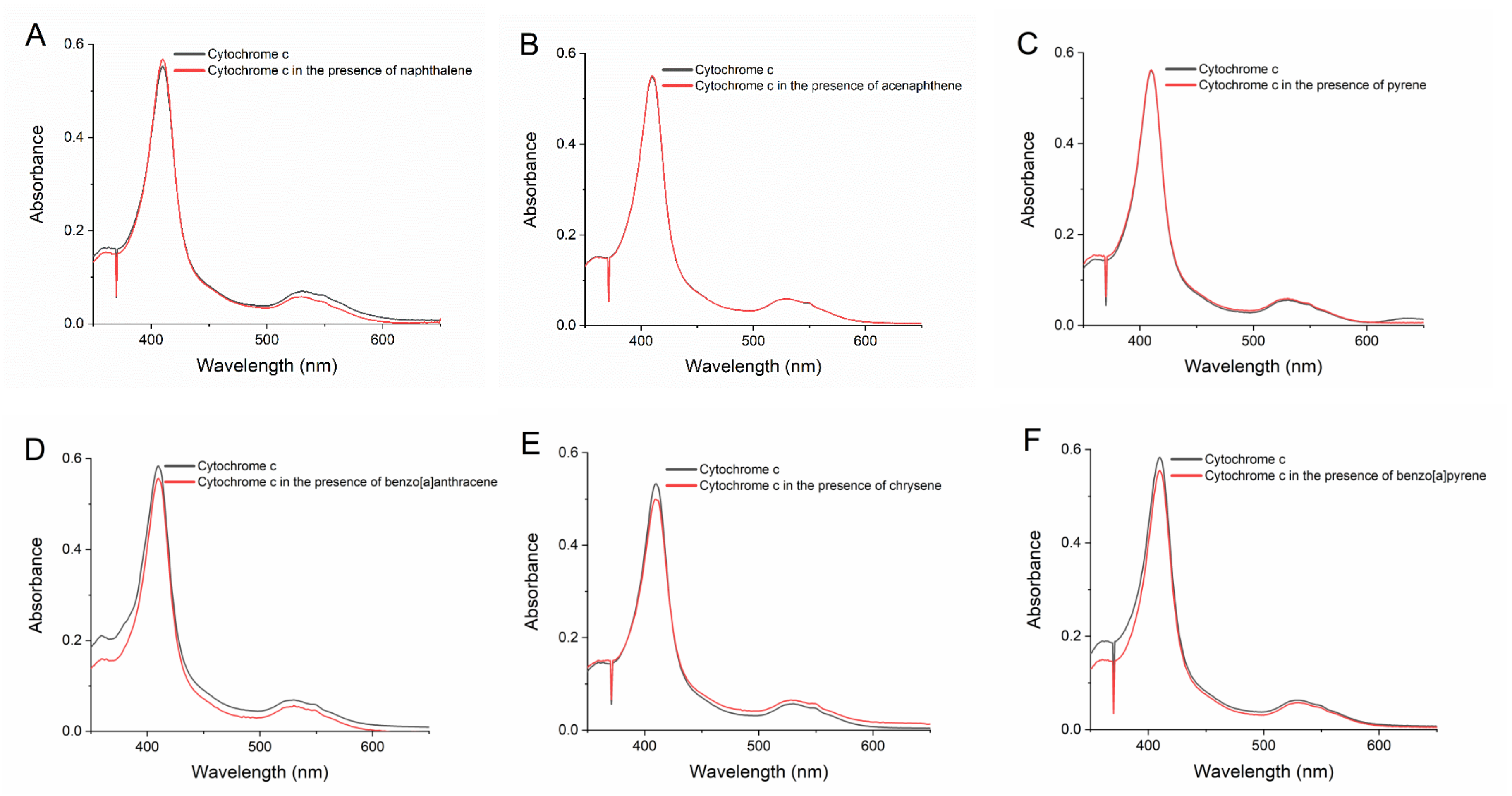

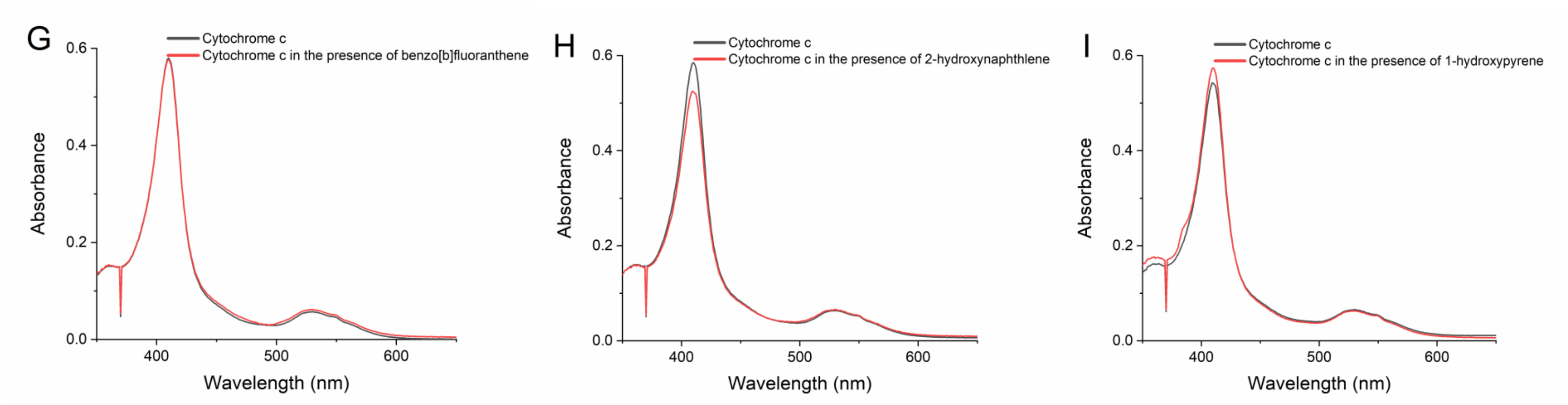

G
Cytochrome c
Cytochrome c in the presence of benzo[b]fluoranthene
Absorbance
Wavelength (nm)
H
Cytochrome c
Cytochrome c in the presence of 2-hydroxynaphthlene
Absorbance
Wavelength (nm)
I
Cytochrome c
Cytochrome c in the presence of 1-hydroxypyrene
Absorbance
Wavelength (nm)
